# Randomised controlled trial of gabapentin in Complex Regional Pain Syndrome type 1 [ISRCTN84121379]

**DOI:** 10.1186/1471-2377-4-13

**Published:** 2004-09-29

**Authors:** Anton C van de Vusse, Suzanne GM Stomp-van den Berg, Alfons HF Kessels, Wim EJ Weber

**Affiliations:** 1 Pain Management and Research Centre, Dept. of Anesthesiology, *Dept. of Clinical Epidemiology and Medical Technology Assessment, Dept. of Neurology, University Hospital Maastricht, Maastricht, The Netherlands

## Abstract

**Background:**

Complex Regional Pain Syndrome type one (CRPS I) or formerly Reflex Sympathetic Dystrophy (RSD) is a disabling syndrome, in which a painful limb is accompanied by varying symptoms. Neuropathic pain is a prominent feature of CRPS I, and is often refractory to treatment. Since gabapentin is an anticonvulsant with a proven analgesic effect in various neuropathic pain syndromes, we sought to study the efficacy of the anticonvulsant gabapentin as treatment for pain in patients with CRPS I.

**Methods:**

We did a randomized double blind placebo controlled crossover study with two three-weeks treatment periods with gabapentin and placebo separated by a two-weeks washout period. Patients started at random with gabapentin or placebo, which was administered in identical capsules three times daily. We included 58 patients with CRPS type 1.

**Results:**

Patients reported significant pain relief in favor of gabapentin in the first period. Therapy effect in the second period was less; finally resulting in no significant effect combining results of both periods. The CRPS patients had sensory deficits at baseline. We found that this sensory deficit was significantly reversed in gabapentin users in comparison to placebo users.

**Conclusions:**

Gabapentin had a mild effect on pain in CRPS I. It significantly reduced the sensory deficit in the affected limb. A subpopulation of CRPS patients may benefit from gabapentin.

## Background

Complex Regional Pain Syndrome type one (CRPS I) or formerly Reflex Sympathetic Dystrophy (RSD) is a disabling syndrome, in which a painful limb is accompanied by varying symptoms like edema, hyperhidrosis, hypertrichosis, allodynia, coloring of the skin and, over time, atrophy of the involved tissue. Spontaneous recovery does occur and several therapies have been described, but for some patients CRPS I is a chronic disabling disease[1]. Neuropathic pain is a prominent feature of CRPS I, occurring in 75% of cases[1], and many researchers go as far as classifying CRPS I as a neuropathic pain syndrome [2-6]. Gabapentin (Neurontin^®^, Pfizer) is an anticonvulsant with a proven analgesic effect in various neuropathic pain syndromes [7-15]. Anecdotal reports suggest that gabapentin may also be an effective analgesic in CRPS patients[3,8,16-27]. To study this hypothesis, we conducted a double blind, placebo-controlled crossover trial of gabapentin in 58 patients with Complex Regional Pain Syndrome type I.

## Methods

### Study population

This study complied with the Declaration of Helsinki regarding investigations in humans after approval of the protocol by the Institutional Review Board of the University Hospital Maastricht, the Netherlands. Patients were recruited from a database with patients who, in recent years, had been diagnosed with complex regional pain syndrome type I in our hospital. All patients had been treated in our pain management and research center (dept. of Anesthesiology, University Hospital Maastricht, The Netherlands) and had received sympathetic blocks[28], mannitol infusions[29,30], and transcutaneous neuromodulation[31]. All participating patients fulfilled the IASP criteria[32] for the diagnosis of CRPS type I and were included if they were between 18 – 75 years old and had a score for pain > 3, as rated on a visual analog score (VAS), where 0 is no pain and 10 is the worst pain imaginable. Apart from IASP criteria, all patients had functional loss and pain outside the original traumatized area. Patients were excluded in case of a possibility of health risk or confounding by other diseases of syndromes, like e.g., pregnancy, known kidney and/or severe liver disease, another (2nd) chronic pain syndrome, known nerve damage in the affected area, active infection or diabetes mellitus. Patients were participating in 8-week periods from 19-11-1998 until 2-12-1999. Gabapentin was not registered as a drug in the Netherlands before or during the trial. After the trial the producing pharmaceutical company supplied gabapentin for compassionate use if indicated.

### Treatment

Since our patient population consisted of chronic CRPS I patients with a multiple years' duration of pain complaints refractory to various treatments, we assumed that their pain complaints would be more or less stable. We therefore undertook a double blind, randomized crossover study. Randomization of patients took place after baseline measurements and written informed consent. The assignment scheme was generated by our hospital pharmacy from a table of random numbers. The closed envelopes containing the assignments were prenumbered and kept at the pharmacy. The first treatment group received gabapentin, followed by a washout period and placebo treatment. The second treatment group received placebo treatment, which was followed by a washout period and gabapentin treatment. Each medication period lasted three weeks separated by the two-weeks washout period. Medication was stocked and delivered to the patient at the hospital pharmacy. Both the gabapentin capsules and the identical placebo capsules were delivered immediately before the start of the two medication periods. Left over medication was recollected and counted.

The gabapentin (GBP) dose was slowly increased to reduce adverse side effects:

600 mg's GBP AN once a day on day 1–2

600 mg's GBP b.i.d. on day 3–4

600 mg's GBP t.i.d on day 5–21

Placebo dose was identically titrated. Patients were allowed to take their usual analgesics and were told preferably not to change the usual dose.

### Follow-up measurements

The patients were reevaluated at the hospital three weeks (T1), five weeks (T2) and eight weeks (T3) after randomization. During the trial, the patient noted her/his pain rate of the past 24 hr (VAS) and the use of additional analgesics in a diary. During each hospital visit the following assessments were done:

1. Global perceived effect (GPE) on pain indicating: worst ever; much worse; somewhat worse; not improved/not worse; somewhat improved; much improved and best ever. GPE on function was scored on an analogous scale.

2. Neuropathic pain scale (NPS), a 10 item qualitative evaluation of neuropathic pain[33].

3. Sensibility through Von Frey monofilament skin application each on 9 areas corresponding to cutaneous nerve branches and dermatomes of either both hands or both feet[34]. Stimulus placement of filaments was as follows: one second for placement, one second for bending and one second for removal. (handset with resp. 0.0677, 0.4082, 2.052 and 3.632 grams calculated force, North Coast Medical, Inc., San José, USA).

4. Mechanic allodynia test with brush strokes and static pressure with the finger tip[35], on 9 areas corresponding to cutaneous nerve branches and dermatomes of either both hands or both feet.

5. Edema, discoloration, and changed skin temperature were scored after physical examination on a three point scale indicating no, some or overt presence of each sign, the latter two signs in comparison to the healthy or healthiest limb. Physical examination in CRPS is well comparable to instrumental evaluation of signs with volumeter, infrared thermometer and goniometer[36].

6. Impairment and disability tests: Symptom Checklist-90-Revised (SCL-*90*-R)[37], Brief Pain Inventory[38], adapted for CRPS to measure the influence of CRPS in general on daily life by 0–10 scale ranging from 0 ('CRPS has not interfered') to 10 ('CRPS completely interfered'), 'range of motion' as a parameter of limb function.

### Side effects during treatment

A blinded independent investigator (STvdB) did sensibility, allodynia and range of motion tests (see above). A physician (AvdV), who examined each patient, did all the other measurements throughout the trial. Patient, investigator and physician were unaware of the treatment received. We tested blinding by questioning physician and participants after each medication period.

### Statistical analysis

The statistical analysis of VAS-scores was determined per patient using estimating medication and period effect through linear regression analyses. Possible relationship of patient characteristics and outcome was tested by Pearson R's test. Mann-Whitney analyses were used for monofilament sensitivity on log-transformed data. Three point scales and seven point scales were dichotomized and like the SCL-90-R, NPS and CRPS-Brief Inventory questionnaires intra-individual paired tested (McNemar, t-test, Bonferroni-Holm corrected for multiple tests). Student t-tests and regression analysis were used to test treatment effect, which is calculated in crossover studies as ((AT1-AT0)-(AT3-AT2))/2+((BT3-BT2))-BT1-BT0)/2, where A represents data of placebo starters and B data of gabapentin starters both before (T0,T2) and after (T1,T3) treatments[39]. Blinding was tested with Chi-square analyses. Possible related factors to therapy effect were analyzed with forward stepwise logistic regression. Data analyses required complete data sets. Patients who were not completing one or two treatments were excluded for analyses. We tested two-tailed, with α = 0.05 as a level of significance (Excel 2000, SPSS 10.0 for Windows).

## Results

### Demographics

**Figure 1:**
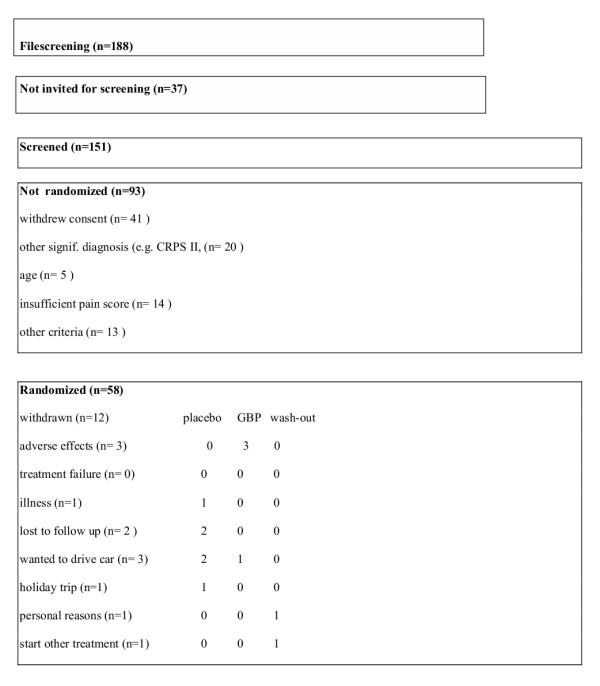
Selection of patients participating in the trial

After randomization 58 patients were enrolled, with a mean age of 44.0 (range 24–75) resulting in 29 patients in the gabapentin-placebo arm and 29 patients in the placebo-gabapentin arm; 49 patients completed the gabapentin period, 50 patients the placebo period, 46 patients completed both periods and were used for further within-patient paired analysis (Fig. 1). Twelve patients discontinued treatment of which 6 during the placebo treatment, 2 during washout and 4 during GBP treatment. Three of these four GBP users discontinued due to side effects (Fig. 1). Between randomization and start of (placebo) medication one patient withdrew after rereading the information letter about possible side effects. These patients were excluded from analysis, since intra-individual testing was necessary for most of the data-analyses. Patients, who could not be used for analysis, did not differ in their characteristics from the total group nor comparing between the two arms of treatment (Tables 1 and 2). When comparing the placebo-GBP arm and GBP-placebo arm on sexes, age and pain level before period 1 or 2, duration of illness, SCL-90-R score, we could not find a difference between the two arms (Tables 1 and 2). SCL-90-R score revealed increased values on any subscale comparing to standard norms, indicating personal distress (Table 2). We found relative higher scores on somatic and sleeping complaints. The SCL-90-R scores were identical to control chronic pain patients (N = 143), besides higher score on sleeping complaints (T. Forouzanfar, data not published). Trial medication was returned and counted afterwards, but revealed no lack of compliance in any patient.

**Table 1:** Patient characteristics

	**Placebo starter**	**GBP starter**		
			Excluded from analysis	

			**Placebo starter**	**GBP starter**

	N = 24	N = 22	N = 5	N = 7
Sex (F/M)	21/4	18/4	3/2	6/1
Age in years	42 (± 13)	47 (± 14)	40 (± 11)	43 (± 11)
Duration in months	43 (± 36)	44 (± 21)	83 (± 39)	45 (± 30)
VAS0	64.2 (± 16)	62.5 (± 18)	62 (± 10)	67 (± 12)
VAS2	67 (± 20)	64(± 21)		
Upper extremity in pain	3R 8L 3RL = 14	8R 7L 4RL = 18	2R 0L 1RL	2R 4L 0RL
Lower extremity in pain	2R 7L 4RL = 13	3R 3L 0RL = 6	2R 1L 0RL	1R 1L 1RL

R/L/RL represents no. of patients that report pain in resp. right, left or bilateral extremities.

A few patients had upper and lower extremity pain. VAS is pain level on visual analogue scale. VAS0 is day 1, VAS2 is day 21 (post wash-out). Data are mean with (SD)

**Table 2:** Basic characteristics of participating patients on neuropathic pain scale (NPS), CRPS brief inventory and SCL-90-R. Data are mean with (± standard deviation).

**NPS**	**Intensity**	**Sharpn.**	**Hot**	**Aching**	**Cold**	**Sens.**	**Itch.**	**Comfo.**	**Deep p.**	**Superf.P.**
Mean N = 24	7,3 (± 1,8)	7,3 (± 1,5)	6,0 (± 3,2)	7,0 (± 2,4)	6,0 (± 3,2)	6,6 (± 2,5)	2,8 (± 2,5)	7,8 (± 1,6)	7,6 (± 1,3)	6,0 (± 2,7)
Mean N = 22	7,3 (± 1,4)	7,4 (± 1,5)	5,9 (± 3,1)	7,2 (± 1,6)	5,9 (± 3,1)	7,2 (± 2,5)	3,7 (± 3,1)	7,7 (± 1,4)	7,8 (± 1,2)	6,8 (± 2,4)
Lost ABN = 5)	7,6 (± 0,5)	8 (± 1,2)	6,2 (± 3,8)	6,8 (± 1,8)	8,2 (± 0,8)	8,4 (± 0,9)	2,8 (± 3,8)	7,3 (± 1,7)	8,2 (± 1,3)	7,4 (± 1,1)
Lost N = 7	7,7 (± 1,4)	7 (± 1,3)	5,7 (± 3,3)	8,3 (± 1,0)	8,4 (± 1,7)	8,6 (± 1,1)	2,3 (± 2,9)	8,8 (± 1,0)	8,3 (± 1,1)	8,6 (± 0,8)
**SCL-90-R**	**anxiety**	**fobic**	**depression**	**somatiz**	**Obs-comp**	**sensitivity**	**hostility**	**insomnia**	**psneu**	

Total AB (n = 24)	15,9 (± 5,8)	10,9 (± 4,0)	31,1 (± 11,4)	26,2 (± 8,3)	19,2 (± 7)	26,8 (± 10,1)	10,2 (± 5,4)	10,1 (± 3,9)	163,0 (± 47,5)	
Total BA (n = 21)	15,9 (± 5,8)	11,2 (± 6,3)	33,6 (± 14,4)	25 (± 8)	19,7 (± 6,0)	28,1 (± 10,5)	8 (± 1,9)	10,3 (± 3,5)	163,8 (± 44,9)	
Lost AB (n = 5)	18,4 (± 11,2)	13,4 (± 8,3)	27,8 (± 10)	26,8 (± 7,8)	21 (± 2,9)	26 (± 2)	9,2 (± 2,8)	11,6 (± 2,1)	166,8 (± 41,2)	
Lost BA (n = 6)	19,7 (± 11,1)	12,2 (± 4,4)	33 (± 17,5)	29,17 (± 11,5)	22,3 (± 9,8)	33,5 (± 18,0)	11,7 (± 7,5)	12,8 (± 2,1)	189,7 (± 80,1)	
**CRPS brief**	**inventory=**	=	=	=	=	=	=	=	=	=
	1	2	3	4	5	6	7	8	9	10
Mean N = 24	7,4 (± 1,7)	6,5 (± 2,1)	6,3 (± 3,1)	7,6 (± 2,0)	4,2 (± 2,6)	7,0 (± 2,8)	6,4 (± 2,2)	4,3 (± 3,4)	7,2 (± 2,2)	5,6 (± 2,5)
Mean N = 22	7,0 (± 2,0)	5,0 (± 3,0)	6,3 (± 3,0)	7,8 (± 2,3)	4,8 (± 2,9)	7,7 (± 2,1)	5,1 (± 3,1)	5,1 (± 2,7)	6,8 (± 2,5)	5,5 (± 2,5)
Lost AB n = 5	7,4 (± 1,8)	7,2 (± 1,6)	7,8 (± 0,4)	9 (± 1)	4,6 (± 1,5)	8,2 (± 0,8)	6 (± 2,6)	6,4 (± 2,6)	7,2 (± 3,6)	6,8 (± 1,1)
Lost BA n = 7	8,6 (± 1,4)	6 (± 3)	6,4 (± 3,9)	9 (± 1,2)	5 (± 2,9)	8 (± 1,6)	7 (± 2,8)	6,7 (± 1,8)	7,3 (± 2,3)	6,6 (± 2,4)

***NPS ****description of pain in terms of 1. intensity 2. sharpness 3. hot 4. aching 5. cold 6. sensitive 7. itching 9. comfortability 10a. intensity deep pain 10b. intensity superficial pain. Item 8 is a nominal scale left out of analysis.*

***Symptom Checklist-90-Revised (SCL-90-R) ****subscale on anxiety, phobic anxiety, depression, somatization, obsessive compulsive, interpersonal sensitivity, hostility, insomnia and psycho neuroticism.*

***CRPS BI***: *influence of CRPS on 1. general activity 2. mood 3. mobility 4. normal work 5. personal relationships 6. sleep 7. enjoyment of life 8. self care 9. recreational hobbies 10. social activities. CRPS BI and NPS on a 0–10 scale*

### Blinding

After each medication period both patient and physician were asked about their ideas concerning study medication in the past period. The treating physician guessed the used medication correctly more often after both phases than can be explained by coincidence (p = 0.000). Blinding for patients was sufficient in the first phase, but not anymore after the second phase (p = 0.2 versus p = 0.000).

### Response to treatment

#### Pain

**Figure 2:**
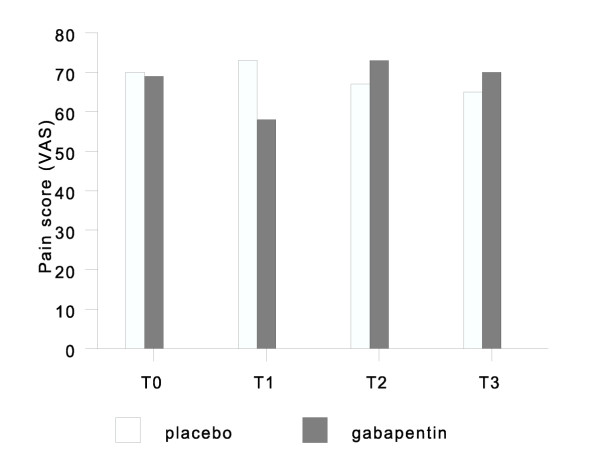
VAS for pain in both groups at start (T0), three weeks (T1), five weeks (T2), and eight weeks (T3) after randomization. T0-1 is the first treatment period, and T2-3 the second

Comparing gabapentin and placebo users in terms of pain relief, there was a significant pain relief in favor of gabapentin in the first period. Therapy effect in the second period was less, finally resulting in no significant effect combining results of both periods. There was an unexpected increase of pain level above baseline in the washout period for both the gabapentin starters and placebo starters (Figure 2).

**Figure 3:**
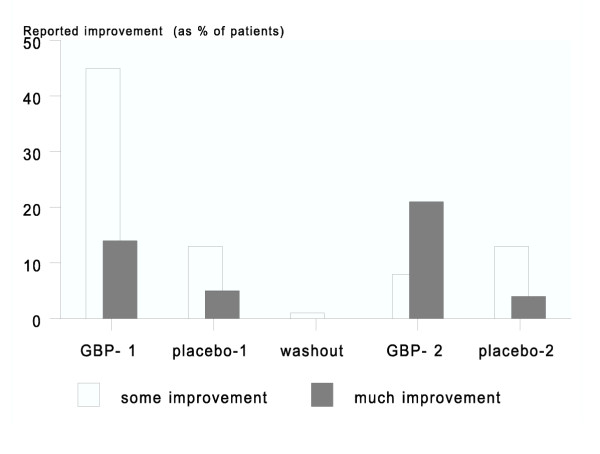
Global perceived pain relief (on a seven-point scale) as reported by patients. GBP-1 and -2 denote patients receiving GBP in the first and second period; placebo-1 and -2 are analogously denoted.

Global perceived pain relief as measured by the seven-point scale showed a significant effect for gabapentin, and also more pronounced in the first period. This measurement also found a significant effect in the second period, with an effect being defined as a patient scoring 'much improvement'. Statistical analysis of global perceived effect showed significant more treatment effect (p = 0.002) with 43 % versus 17 % reported pain relief respectively during gabapentin compared to placebo treatment. 13 % of patients reported aggravation of pain during gabapentin vs. 9 % during placebo treatment (Figure 3 and table 3). Stepwise forward logistic regression analysis of baseline value of pain level, age, sex, duration of illness, location of illness, mono- or bilateral CRPS, trial arm and all items of CRPS-BI, NPS and SCL-90-R was performed. Only the level of self care was related to perceived pain relief during GBP. The neuropathic pain scale, indicating different aspect of pain, improved significantly in terms of less hot and more comfortable, but not when corrected for multiple tests (Bonferroni-Holm correction). We found that during gabapentin use, patients reported equal use of co-medication comparing to baseline assessment and placebo-use with a non-significant trend towards less medication during GBP use.

**Table 3:** Patients (%) with global perceived effect on pain in the four arms of treatment and totals for the two treatments.

Treatment period	GBP-1=	Placebo-1=	Wash-out=	GBP-2=	Placebo-2=
% some improvement (n)	45% (10)	13% (3)	1	8% (2)	13% (3)
% much improvement (n)	14% (3)	5% (1)	0	21% (5)	4% (1)
% total (n/N)	59% (13/22)	^a ^17% (4/24)	1	29% (7/24)	18% (4/22)
=	Total = GBP =	=	Total = placebo =	=	=
% some improvement	26% (12/46)		13% (6/46)		
% much improvement	17% (8/46)β		4% (2/46)		
% total (n/N)	43% (20/46)^a^		17% (8/46)		
worsened	13% (6/46)		9% (4/46)		

GBP-1 is gabapentin treatment before wash-out. GBP-2 is gabapentin treatment after wash-out. 'α' is significant, P < 0.005, 'β' is P < 0.10 McNemar two sided tested gabapentin versus placebo.

### Sensory tests

Each participant was tested throughout the study on response to mechanical stimuli with von Frey filaments. The CRPS patients had sensory deficits at baseline. Application of smaller filaments was not felt in multiple skin areas. We found, with Mann-Whitney analyses, that this sensory deficit was significantly reversed in gabapentin users in comparison to placebo users (p = 0.027). This difference was found in patients with upper and lower extremity CRPS, but was still significant in the subgroup of lower extremity CRPS (p = 0.011) as seen in table 4.

**Table 4:** Mann-Whitney scores of monofilament application in CRPS patients testing cutaneous sensibility thresholds

	**Mean ranking**		
	**Hand**	**Feet**	**Total**

**Placebo**	12.0 (N = 12)	5.5 (N = 10)	16.8 (N = 22)
**Gabapentin**	15.6 (N = 15)	12.0*(N = 3)	25.0*(N = 18)

Significant different values (p < 0.05) are marked with*.

Mechanical allodynia to static and dynamic stimuli (soft touch and brush) was measured by a mean of 11-point scales (range 0–10) of 9 areas of the hand/feet corresponding to cutaneous nerve branches. We found no effect of gabapentin on allodynia in comparison to placebo.

### Other symptoms

No difference was found on the parameters edema, discoloration, range of motion of wrist/ankle and fingers/toes between placebo and GBP. 10 patients out of 45 improved in relative skin temperature during placebo use compared to 18 patients out of 45 in gabapentin, which is two sided tested not significantly different (McNemar analysis, p = 0.096).

### Limb dysfunction and quality of life

The reported function improvement was, with 10 positive responders during GBP versus 7 positive responders during placebo, not significantly different (N = 46) between the two treatments. The SCL-90 showed no significantly better scores during gabapentin treatment. CRPS-BI showed improvement of sleep between placebo treatment and gabapentin treatment., but this effect disappeared after Bonferroni-Holm correction.

### Adverse effects

Dizziness, somnolence and lethargy were significantly more often reported during gabapentin use than during placebo. Before washout 95 % of patients (n = 21) reported side effects during gabapentin use versus 58 % in placebo treatment (n = 14). After washout this was respectively 63% (n = 15) in GBP and 32% (n = 7) in placebo use. For more details on side effects see table 6. Since a high incidence of side effects can produce a stronger placebo effect, we analyzed the possible correlation between side effects and pain relief. There was a small relation between perceived side effects and pain relief in placebo users in period 2 (p = 0.04, Pearson's R value is 0.4), but, whether in period 1 or period 2, no relationship was found during the use of gabapentin (p = 0.2 in period 1, P = 0.4 in period 2).

## Discussion

To evaluate gabapentin treatment as a treatment for pain in CRPS, we conducted a placebo-controlled crossover study. We conclude from our trial that overall, gabapentin did not relieve pain as compared to placebo on pain visual analogue scores, our primary outcome measure. Gabapentin relieved pain in a subgroup of patients and gave a significant global perceived pain relief. The effect was mild and there was no patient in which gabapentin completely eliminated pain. Moreover, the frequency of side effects as dizziness, somnolence and lethargy was higher during gabapentin treatment than with placebo. These side effects probably also account for the relative lack of blinding we observed in our study. This does not mean that the study was biased: our population was chronic CRPS patients who all had undergone numerous unsuccessful treatments, and clearly wanted the drug to work. Any possible bias would therefore have been positive towards gabapentin.

Although we did not find a significant pain reducing effect when analyzing the complete trial, we did find a significant effect in the first half of the trial. In fact, the difference in outcome between the two trial halves is striking. There was a reverse carry-over effect resulting in increasing pain above baseline after the washout period for both gabapentin and placebo starters. The increase of pain intensity above baseline level in the second period (before the start of placebo treatment) cannot be explained pharmacologically. Gabapentin has no known biological dependency or tolerance mechanism. It can be a period effect, although this would more likely result in a regression to the middle instead of increasing pain. Perhaps this is a reversed placebo effect in which the expectation and/or the actual perception of not receiving gabapentin anymore might increase pain intensity. Kemler and de Vet found that treatment allocation in a trial could influence pain intensity in CRPS[40]. The decreasing therapy effect after washout is found in other crossover pain trials[41]. Expectation and attention have been shown to be powerful influences on pain pathways in the brain[42], and perhaps a crossover design is not suited to study treatments in chronic pain patients.

We found a decreased sensory deficit in gabapentin users compared to placebo users. We did not expect this, but found in the literature several cases in which gabapentin decreased the area of hypesthesia in neuropathic pain syndromes[43]. This has, to our knowledge, never been described for any other medication. Numbness or mechanical hypesthesia is a frequently found complaint for approximately 75 % of CRPS patients, which can improve in time spontaneously and after placebo treatment[44]. It is possible that the somatosensory findings and pain outside the original area of trauma can be attributed to referred pain mechanisms. Gabapentin has been reported to alleviate referred pain[45]. Since many CRPS patients have mechanical hypesthesia, we hypothesize that gabapentin influences some common neural pathway for 'referred' sensations, whether mechanical sensation or pain.

## Conclusions

Gabapentin had a mild effect on pain in patients CRPS I. It significantly reduced the sensory deficit in the affected limb. A subpopulation of CRPS patients may benefit from gabapentin, but then for each individual patient the benefit has to be weighed against the frequently occurring side effects.

## Competing interests

Parke-Davis (now a Warner-Lambert/Pfizer division) supplied gabapentin and matching placebo capsules for this trial. Drs. Van de Vusse and Weber have received financial support from Parke-Davis to attend one congress. Parke-Davis has had no role in the writing of this manuscript

## Authors' contributions

AvdV initiated the trial and wrote, with WEJW and AHFK, the protocol. The study and its data management was done by AvdV and SS-vdB. AHFK did the statistical analyses. AvdV wrote the first draft of the manuscript, which was finished in its final form by WEJW.

**Table 5:** Side effects as mentioned after treatment

**Adverse effect**	**Gabapentin (N = 54) n (%)**	**Placebo (N = 51) n(%)**	**Significance**
Dizziness	20 (37.3)	2 (3.9)	P = 0.0000
Somnolence	15 (27.8)	3 (5.9)	P = 0.003
Lethargy	11 (20.4)	1 (2.0)	P = 0.003
Nausea	10 (18.5)	5 (9.8)	n.s.
Headache	8 (14.8)	3 (5.9)	n.s.
Stomach problems	4 (7.4)	3 (5.9)	n.s.
'drunken'	4 (7.4)	0 (0)	n.s.
Disturbed gait	4 (7.4)	0 (0)	n.s.
Water retention	1 (1.9)	3 (5.9)	n.s.

Data on all patients who started treatment and returned for assessment after 3 weeks, with or without completing 3 weeks of treatment. n.s. is 'not significant'

## Pre-publication history

The pre-publication history for this paper can be accessed here:


